# Pictorial Health Warning Labels on Cigarette Packages: An Investigation on Opinions of Male Smokers

**DOI:** 10.5812/ircmj.14879

**Published:** 2014-03-05

**Authors:** Davoud Shojaezadeh, Nooshin Peyman, Mohammad Taghi Shakeri, Saharnaz Nedjat, Abbas Mohaddes Hakkak, Mohammad Hossein Taghdisi, Hamid Reza Mohaddes Hakkak, Keivan Shariati, Ali Taghipour

**Affiliations:** 1 Department of Health Education and Promotion, School of Public Health, Tehran University of Medical Sciences, Tehran, IR Iran; 2 Department of Health and Management, School of Health, Mashhad University of Medical Sciences, Mashhad, IR Iran; 3 Department of Biostatistics and Epidemiology, School of Health, Mashhad University of Medical Sciences, Mashhad, IR Iran; 4 Department of Epidemiology and Biostatistics, School of Public Health, Knowledge Utilization Research Center, Tehran University of Medical Sciences, Tehran, IR Iran; 5 Department of Rehabilitation, Behzisti (Elmi-Karbordi) University of Mashhad, Mashhad, IR Iran; 6 Department of Health Education and Promotion, School of Public Health, Iran University of Medical Sciences, Tehran, IR Iran; 7 Department of Biostatistics and Information Technology, School of Nursing and Midwifery, Mashhad University of Medical Sciences, Mashhad, IR Iran

**Keywords:** Smoking, Tobacco Products, Iran

## Abstract

**Background:**

Health warning labels on cigarette packages are among the most straightforward and important tools to communicate with smokers and various studies have illustrated their efficacy.

**Objectives:**

The current study aimed to investigate the opinions of male smokers in Mashhad city about the efficacy of health warning labels printed on cigarette packages on the smoking status of smokers.

**Patients and Methods:**

This cross-sectional descriptive study was conducted in 2013 using a questionnaire. The research population included the male smokers of Mashhad. The participants were selected from the customers referring to the newsstands for cigarettes. The obtained data were analyzed employing SPSS software Version 16, and the statistical tests including Kruskal-Wallis, Spearman, and correlation coefficient of Pearson, Chi Square, Mann-Whitney, and Bonferroni correction were used in this regard.

**Results:**

In this research, there were 500 participants with the average age of 25 years. The initiation age of smoking was eight years while the maximum age was reported as 45 years. Results of this research about the effect of these labels on decreasing cigarette consumption rate showed that almost half of the participants believed that these labels were ineffective for them (52.2%) and other smokers (53.8%).Furthermore, significant relationship was found between the age and opinion of the smokers about the influence of these labels on reducing their cigarette consumption (P < 0.001).

**Conclusions:**

To promote the effect of printed images on cigarette packages, it is recommended to consider the suitability of labels in the targeted culture. In addition, to be more effective consultation sites to quit smoking should be introduced under the images.

## 1. Background

According to the World Health Organization (WHO) reports, every six seconds, one person dies in the world because of the side effects of smoking (1). It is predicted that in 2030, smoking would be the cause of death for above eight million people throughout the world (2). Currently, the governments and health organizations are conducting a large number of programs to reduce smoking rate in the society. In this regard, one of the most effective ways to prevent , or even quit smoking is using health warning labels on cigarette packages (3). In 1996, the USA took some actions for the first time by printing health warnings on cigarette packages (4). From that date on, many countries have applied similar measures (5). Since most countries have banned or limited the public advertisement of cigarettes, to advertise and communicate with their present and potential customers tobacco industries have mainly focused on the design of cigarette packaging (6). This is why Article 11 of the framework convention tobacco control (FCTC) introduces using health warning labels on cigarette packages among the strategies to prevent smoking, and encourage quitting (7). Indeed, FCTC is based on the principle that all people should be aware of the unpleasant consequences, addicting nature, and fatal dangers of smoking (8).

Health warning labels on cigarette packages are among the most straightforward and important tools to communicate with smokers (9), and various studies have illustrated their efficacy (9-11). Iran, with the smoking rate of 12.5% (12), has officially started printing health-warning labels on cigarette packages from 2009 (13). Owing to the fact that, by now, no comprehensive study has been conducted to examine the effect of cigarette package labels on smokers in Iran, there is no thorough information available in this regard. Therefore, the present study was performed on male smokers in Mashhad, as the second largest city of Iran to investigate their opinions on the efficacy level of health warning labels.

## 2. Objectives

The current study aimed to investigate the opinions of male smokers in Mashhad about the efficacy of health warning labels printed on cigarette packages on the smoking status of smokers. The primary objectives of the current study include investigating the smokers` opinion about the effect of the labels on reducing cigarette consumption rate in them or other smokers, as well as the effect of the labels on giving up smoking in them and other people.

## 3. Patients and Methods

This cross-sectional descriptive study was conducted in 2013 using a questionnaire.

### 3.1. Participants

The research population included male smokers of Mashhad city, Iran. Considering the fact that according to the previous studies the population of male smokers (17.2%) outnumbers that of the female smokers (2.5%) (14), and due to the fact that in the Iranian culture, smoking is regarded as a stigma for women, and obtaining reliable information is somewhat difficult (12), the samples were selected only from male smokers. Inclusion criteria for this study were being male, being smoker, and willing to participate in the research, while the exclusion criterion was just reluctance to participate. Newsstands which sold cigarettes were selected as the sampling sites. The participants were selected from the customers referring to the newsstands for cigarettes.

### 3.2. Ethical Considerations

After being provided with the necessary information about the objectives of the research, the participants were asked to study and sign the informed consent letters after briefing about the study objectives, and were assured about the confidentiality of their information. There were no voice or video recording during the interviews, and the participants` responses were written in the questionnaires by the interviewers. Ethical considerations and ethical codes of this research were approved by the Elmi-Karbordi University (under 17002-118 dated 18/1/92).

### 3.3. Sampling Method

To select the samples, the multistage method was applied, similar to the other studies (15). In accordance with the map of Mashhad city and municipality divisions, the central district of Mashhad was selected. Since the central part of Mashhad is a representative of the overall city in terms of various parameters, and because of its greater area and population diversity, it was selected as the sampling area. The central district of Mashhad is divided into four main zones, based on urban divisions. One area was randomly selected from each zone. Then, from every area, one main street, and from every main street, one cigarette stand was randomly selected. Totally, four newsstands were selected for data gathering and sampling locations. Then the samples were selected from the customers referring to the cigarette stands, as an accessible sample. Based on the formula to measure sample size, 83 samples had to be selected from each location that totally, 333 samples were considered as required samples (Equations 1 and 2).

Equation 1. $$n=\frac{{\left(Z_{1-\frac{\alpha }{2}}+Z_{1-\beta }\right)}^{2}(s_{1}^{2}+s_{2}^{2})}{d^{2}}$$

However, due to the possibility of access to more samples for researchers and to achieve the quorum of 83 people in all sampling points, the sampling continued until the tenth day. Consequently, the number of samples was more than the predicted sample size. The sampling process continued until 500 subjects were interviewed. During 10 days of sampling in all of the sampling areas, 575 people were included in the study. However, 75 people were excluded because of their lack of interest to continue the interview, answer all of the questions, and sign the consent letter.

Equation 2 $$n=\frac{{\left(1.96+1.64\right)}^{2}(80+80)}{25}≃83$$

### 3.4. Data Collection

The required data were gathered during the interviews using a questionnaire. To design the questionnaire, similar studies that the validity and reliability of their questionnaires had been confirmed, were studied and a new questionnaire was designed (15). The questionnaire consisted of 13 questions. The first part of the questionnaire included some demographic information of the participants including their age, level of education, and the onset age of smoking. The second part contained questions about the people’s perspective on efficacy of health warning labels on cigarette packages regarding the reduction of cigarette use and even quitting, on them and other smokers. The answers to these questions were designed as very much, much, average, little, very little, and ineffective. The questions of the third part included the number of quits, number of cigarettes consumed in a day, cigarette supply method, and preference to use cigarettes with or without labels. Due to the fact that the people who were buying cigarettes did not have much time to respond to the questions, the questionnaire had been designed in a way that it only took 5 to 10 minutes to fill. To determine the validity of the questionnaire, an expert panel was used in this study (CVI = 94%). Besides, to determine reliability of the questionnaire, correlation of the questions were tested and confirmed using the pilot test and Cronbach’s Alpha (α = 0.912). To minimize the interviewers` errors, three training and coordination sessions were held. Eight interviewers gathered the information in morning and evening sessions. Since the data were collected through interviews, the researchers received the answers to all of the questions at the time of interviews, and there were no missing data. Incomplete questionnaires were excluded from the study.

### 3.5. Analysis

The obtained data were analyzed using SPSS software version 16, through the statistical tests including Kruskal-Wallis, Kendall tau-b correlation coefficient, Chi square, Mann-Whitney, and Bonferroni correction. 

## 4. Results

In the current research, 500 subjects, with the average age of 25 years, participated. The onset age of smoking was eight years while the maximum age was reported as 45 years. The minimum and maximum number of cigarette use per day was reported as 2 and 40 per day, respectively (Table 1). Results of the current study revealed that 68.4% of the participants believed that the warning labels were ineffective on the quitting state of other smokers, while merely 2.4% of them mentioned the efficacy of average to very much of these labels. However, the majority (60.8%) of the participants mentioned that these labels were ineffective on their own quitting state, while the rest of the participants evaluated the effect of these labels as little (25.4%), average (4.8%), and much (8%), respectively (Table 2). 

**Table 1.** Descriptive Specifications of the Study Participants (n = 500)

	Mean ± SD	Minimum	Maximum
**Age, y**	25.03 (9.12)	14.00	58.00
**Onset age of smoking, y**	17.43 (5.38)	8.00	45.00
**Smoking duration, y**	7.53 (7.35)	0.10	38.00
**Average number of daily cigarette use**	10.12 (8.53)	2.00	40.00
**The number of quits**	1.33 (1.6)	0.00	8.00

**Table 2.** Frequency and Frequency Rate of the Responses to the Questionnaire

Question	Results, No. (%)
**Effect of images on quitting of other smokers**	
Very much	4 (0.8)
Much	2 (0.4)
Average	6 (1.2)
Little	21 (4.2)
Very little	125 (25)
Ineffective	342 (68.4)
**Effect of images on decreasing the cigarette use of other smokers**	
Very much	3 (0.6)
Much	8 (1.6)
Average	17 (3.4)
Little	57 (11.4)
Very little	146 (29.2)
Ineffective	269 (53.8)
**Reporting the quit case in acquaintance by observing the warning images**	
Yes	58 (11.6)
No	442 (88.4)
**Preference of either imageless or with image packages**	
With image	40 (8.0)
Without image	315 (63.0)
Indifferent	145 (29.0)
**Effect of images on quitting of himself (respondent)**	
Very much	0 (0.0)
Much	4 (0.8)
Average	24 (4.8)
Little	41 (8.2)
Very little	127 (25.4)
Ineffective	304 (60.8)
**Effect of images on decreasing the cigarette use of himself (respondent)**	
Very much	4 (0.8)
Much	12 (2.4)
Average	29 (5.8)
Little	59 (11.8)
Very little	135 (27.0)
Ineffective	261 (52.2)
**Reporting the cigarette use case in acquaintance by observing the warning images**	
Yes	167 (33.4)
No	333 (66.6)
**Cigarette supply ways**	
Always in packages	108 (21.6)
Mostly in packages and occasionally in number	61 (12.2)
Mostly in number and occasionally in packages	64 (12.8)
Always in number	267 (53.4)

Results of the current research regarding the effect of these labels on decreasing cigarette consumption rate showed that almost half of the participants believed that these labels are ineffective for them (52.2%) and other smokers (53.8%). Besides, 11.6% of them mentioned that they had seen at least one person who was influenced by these labels and quit smoking while the rest of them (88.4%) did not report such cases. Besides, some of the participants mentioned that they had seen some smokers influenced by these labels had decided to reduce their cigarette use; however, 66.6% of them did not report such a case. By means of performing the Kolmogorov-Smirnov test, normality of distribution of age and average of daily cigarette use were examined and normal distribution of these variables were rejected (P < 0.001). Due to the skewness of the age and number of daily cigarette use, non-parametric Kruskal-Wallis test was performed. According to the data shown in Table 3, analysis of the parameters using the Kruskal-Wallis test indicated that the number of daily cigarette use in smokers correlates with the influence level induced in them by these labels (P < 0.001). Furthermore, significant relationship was found between the age and opinion of the smokers about the influence of these labels on reducing their cigarette consumption (P < 0.001). Using the Mann-Whitney and Bonferroni tests, a dual comparison was conducted among the significant items shown in Table 3. The results of this test revealed that the age of people who selected the little option with those who selected very little option significantly varies when responding to the influence of these labels on reducing cigarette consumption (P < 0.001). Moreover, this difference was also significant for “little” and ineffective responses (P < 0.001).

**Table 3.** Comparison of the Responses for Each Question With the Average Age and With the Average Number of Daily Cigarette Use ^a^

	Age		Average Number of Daily Cigarette Use	
	Mean ± SD	P Value	Mean ± SD	P Value
**Effect of image in cigarette quit in other smokers**		0.174		0.130
Very much	24.75 ± 6.95		5.00 ± 2.45	
Much	25.50 ± 0.71		12.00 ± 4.24	
Average	31.33 ± 9.52		8.17 ± 6.05	
Little	27.14 ± 9.15		10.14 ± 5.88	
Very little	25.81 ± 9.14		10.42 ± 7.45	
Ineffective	24.51 ± 9.12		10.09 ± 9.12	
**Effect of image in cigarette use decrease in other smokers**				0.001
Very much	22.00 ± 5.20	> 0.001	4.00 ± 1.73	
Much	25.00 ± 9.0		9.75 ± 6.25	
Average	27.59 ± 9.55		10.00 ± 5.96	
Little	29.12 ± 8.38		11.25 ± 6.66	
Very little	25.05 ± 9.54		10.77 ± 8.26	
Ineffective	24.02 ± 8.83		9.61 ± 9.21	
**Effect of image in cigarette quit in smokers**				0.455
Very much		0.62		
Much	32.25 ± 7.89		9.25 ± 5.06	
Average	27.79 ± 9.88		9.13 ± 5.75	
Little	25.20 ± 7.70		9.59 ± 5.44	
Very little	25.21 ± 8.47		10.46 ± 8.31	
Ineffective	24.62 ± 9.47		10.14 ± 9.17	
**Effect of image in cigarette use decrease in smokers**				0.024
Very much	29.05 ± 11.12	0.064	9.75 ± 4.27	
Much	25.08 ± 7.98		8.25 ± 3.93	
Average	27.83 ± 8.51		9.76 ± 5.94	
Little	26.00 ± 9.05		10.25 ± 5.67	
Very little	25.22 ± 8.79		10.44 ± 7.64	
Ineffective	24.33 ± 9.36		10.05 ± 9.87	
**Image preference**				0.415
With image	24.78 ± 7.92	0.050	7.58 ± 4.85	
Without image	24.20 ± 8.72		10.30 ± 8.97	
Indifferent	26.91 ± 10.01		10.43 ± 8.26	
**Cigarette supply way**				> 0.001
Always in package	24.69 ± 8.97	< 0.001	22.39± 8.01	
Mostly by packages and occasionally in number	28.25 ± 7.85		13.41 ± 4.67	
In number	28.25 ± 7.85		13.41 ± 4.67	
Mostly in number and occasionally in package	22.72 ± 6.35		8.22± 2.33	
Always in number	4.86 ± 3.49		20.94 ± 6.47	

^a^ Due to the skewness of the age and number of daily cigarette use, non-parametric Kruskal-Wallis test was performed.

**Table 4.** Examination of the Linear Relationship Between Some Studied Parameters Using the Kendal Tau-b Correlation Coefficient

	Age	Onset age of smoking	Smoking duration	Number of daily cigarette use	Influence in other’s quitting the smoking	Influence in daily smoking decrease of the others	Influence in quitting the smoking of the individual	Influence in daily smoking decrease of the individual	Quit by observing the images	Decrease in daily use by observing the images	Number of quits
**Age**	1.000										
**Onset age of smoking**	0.414^a^	1.000									
**Smoking duration**	0.561^b^	-0.072^ a^	1.000								
**Number of daily cigarette use**	0.499^b^	-0.068^ a^	0.730^b^	1.000							
**Influence in other’s quitting the smoking**	-0.086^a^	-0.089^a^	-0.056	-0.077^a^	1.000						
**Influence in daily smoking decrease of the others**	-0.126^b^	-0.107^b^	-0.102^b^	-0.136^ b^	0.584^ b^	1.000					
**Influence in quitting the smoking of the individual**	-0.089^a^	-0.106^ b^	-0.037	-0.065	0.558^ b^	0.576^ b^	1.000				
**Influence in daily smoking decrease of the individual**	-0.103^ b^	-0.100^ b^	-0.075^a^	-0.119^ b^	0.504^ b^	0.709^ b^	0.689^ b^	1.000			
**Quit by observing the images**	-0.142^b^	-0.103^ b^	-0.105^ b^	-0.099^ b^	0.489^ b^	0.445^ b^	0.434^ b^	0.412^ b^	1.000		
**Decrease in daily use by observing the images**	-0.244^b^	-0.129^b^	-0.217^b^	-0.240^b^	0.456^ b^	0.594^ b^	453^b^	0.561^ b^	0.498^ b^	1.000	
**Number of quits**	0.551^b^	0.185^ b^	0.547^ b^	0.417^ b^	-0.057	-0.083^a^	-0.044	-0.037	-0.112^ b^	-0.172^ b^	1.000

^a^ 5% Significance level.

^b^ 1% Significance level.

## 5. Discussion

In the current study, the average age for onset of cigarette use was 17.43 ± 5.38, which was close to the reported age range in other studies performed in Iran (12, 14). Regarding the educational status of the smokers, the research conducted by Boskabady et al. in Mashhad indicated that the majority of the smokers had low educational level (14), which was not consistent with the results of the present study. The average number of daily cigarette use was 10.12. Although this number is close to the number reported by Meysamie et al. (13.7 per day) (12), they are still different. This can be attributed to the fact that they studied smokers from Tehran with higher number of participants (5287 participants). In addition, their study was conducted among both male and female subjects, while the present study is the representative of only male smokers from Mashhad city.

Article 11 of FCTC of the WHO introduces using health warning labels as an effective strategy to decrease cigarette use or even encouraging quitting (7), and several studies have confirmed the efficacy of this method (9, 10, 16, 17). However, the findings of the current research indicate that the majority of the studied cases believed that labels on the cigarette packages had little effect or were ineffective on decreasing their daily cigarette use. Moreover, the participants mentioned that the labels had little effect or were ineffective on decreasing the daily cigarette use of other smokers. Since the images and notations used in these labels were the same as the items used in guidelines of article 11 of FCTC (15), such low influence cannot be necessarily due to the labels design. Nevertheless, it is also suggested to use the experiences of other countries to improve the efficacy of these labels (e.g. using more explanations with more negative themes about the diseases caused by smoking, performed in Brazil) (13). The results of the study performed by Romer et al. revealed that the smokers informed of smoking dangers are more encouraged to quit (18). Moreover, a study conducted in Brazil revealed that about two third of the smokers reported their willingness to quit smoking because of these labels, which is in contrast with the results of the current study (19). The authors of the present study believe that inappropriate messages printed on these warning labels are the reason for their little or no effect on quitting smoking. Once the culture and values of the target society are not considered in designing the labels, their message would lose both efficacy and efficiency (20). Therefore, it is recommended to reassess and revise the images and messages used in such labels. According to the data shown in Table 5, significant relationship was found between responses of the people about effectiveness of these images on cutting their cigarette use or quitting, and responses to their preference of choosing between the packages with or without images (P < 0.001). Here, it must be noted that 303 (64.1%) of the smokers who believed in little effect or ineffectiveness of these warning labels on their quitting, preferred the packages without such labels. Indeed, 96.2% of the people who preferred to buy packages without images stated that these images were with little effect or ineffective on their cigarette use or quitting. This contradiction between their action and opinion might be a defensive mechanism. In other words, people might have an unpleasant feeling about smoking when seeing these warning images, and still use denial mechanism to eliminate such a feeling and enjoying smoking. It is worth mentioning that the denial mechanism is a defensive mechanism through which the individuals defend themselves against anxiety by denying some painful aspects of the reality through denial of the sensory data (21).

**Table 5.** Examining the Relationship Between Preference of Imageless and With Image State of the Packages and the Opinion of the Smokers About its Effect on Their Cigarette Consumption Reduction and Quit ^a^, ^b^

	Preference between imageless and with image packages			P Value
	With image	Imageless	Indifferent	
**Effect of warning image on smoking quit in the individual**				< 0.001
Very much, much, average	13 (32.5)	12 (3.8)	3 (2.1)	
Little, very little, ineffective	27 (67.5)	303 (96.2)	142 (97.9)	
**Effect of warning on decreasing cigarette usein the individual**				< 0.001
Very much, much, average	17 (42.5)	22 (7.0)	6 (4.1)	
Little, very little, ineffective	23 (57.5)	293 (93.0)	139 (95.9)	

^a^ All data are presented in No. (%).

^b^ Chi-square test was performed.

The above mentioned point is also true when responding to the question of how effective are the warning images on the cigarette packages in regard to your cigarette use? In this regard, 62% of the participants who selected items little or ineffective selected packages without images. Considering the fact that only 8% of the interviewees preferred packages with images, it can be assumed that the cigarette packages without labels are more attractive for smokers. The results of the study conducted by White et al. also confirm this hypothesis. In their research, the participants considered the cigarettes of packages with just brand and advertisement more attractive and appealing (22). Considering all the above-mentioned points in this section, such a contradiction between action and opinion can be a research subject for further studies. According to the findings of the current study, it is also recommended to use the experiences of other countries regarding promoting efficacy of the warning images. Some countries have invented and applied innovative approaches for enhancing the efficiency of these images. For example, in New Zealand, a phone number called Quitline is assigned for helping and offering support to those who intend to quit smoking (23). In this regard, the authors of the present study suggest allocating such a number for offering support beside the warning images for those who are willing to decrease their cigarette use or quit smoking. Such call centers respond to the smokers` questions on the side effects of smoking, the manner of quitting, and decreasing their cigarette use. Moreover, call centers can provide the address, specifications, and information of the nearest cigarette-quitting center to their work place or home through these numbers. Furthermore, considering the increasing rate of internet usage in the society, it is possible to provide comprehensive information and recommend interactive websites on quitting alongside the warning labels. Considering the fact that this research was conducted only in the central part of Mashhad using the convenience sampling method, it is recommended to conduct complementary researches using random sampling strategies throughout Mashhad. Among other limitations of this study, ignoring female smokers, lack of considering some demographic information such as (job, economic status, and neighborhood etc.), and limiting the sampling process to a specific site (newsstands) can be also named. It is hoped that other researchers consider these limitations through future studies.
